# Applications of CRISPR/Cas9 as New Strategies for Short Breeding to Drought Gene in Rice

**DOI:** 10.3389/fpls.2022.850441

**Published:** 2022-02-24

**Authors:** Jae-Ryoung Park, Eun-Gyeong Kim, Yoon-Hee Jang, Rahmatullah Jan, Muhammad Farooq, Mohammad Ubaidillah, Kyung-Min Kim

**Affiliations:** ^1^Division of Plant Biosciences, School of Applied Biosciences, College of Agriculture and Life Science, Kyungpook National University, Daegu, South Korea; ^2^Crop Breeding Division, National Institute of Crop Science, Rural Development Administration, Wanju, South Korea; ^3^Department of Agronomy, Faculty of Agriculture, Jember University, Jember, Indonesia

**Keywords:** abiotic stress, agricultural trait, generation, *OsSAP*, reactive oxygen species

## Abstract

Recent unpredictable climate change is the main reason for the decline in rice yield. In particular, drought stress is a major constraint in reducing yield and quality for rice at rainfed agriculture areas, such as Asia and South America. CRISPR/Cas9 provides an effective solution for gene function study and molecular breeding due to specific editing of targeted genome sequences. In addition, CRISPR/Cas9 application can significantly reduce the time required to develop new cultivars with improved traits compared to conventional complex and time-consuming breeding. Here, drought-induced gene *Oryza sativa Senescence-associated protein* (*OsSAP*) was edited by CRISPR/Cas9. To investigate the possible role of *OsSAP* in drought stress, genome-editing plants were subjected to drought stress until the soil moisture content reached 20%, and the reactive oxygen species (ROS) scavenging efficiency of genome-editing plants were decreased. When the genome-editing plants were subjected to drought stress, survival rate, shoot length, root length, content of chlorophyll number of tiller, and 1,000-grain weight decreased, and more H_2_O_2_ and O_2_^−^ were detected in leaves. In addition, expression levels of several critical stress-related transcription factors were decreased in the *OsSAP* genome-editing plant. These results suggest that *OsSAP* function as a positive regulator during drought stress response in rice. We analyzed the expression of *OsSAP* and *Cas9* in T_0_ and T_1_ plants as well as T_2_ seeds. As the course of generation advancement progressed, *Cas9* expression remained stable or weakened but the *OsSAP* expression was continuously removed from the T_0_ plant. The coefficient of variation (CV) in both T_1_ plants and T_2_ seeds was lower than 5%. Overall, our results suggest that CRISPR/Cas9 could be a novel and important tool for efficiently generating specific and inheritable targeted genome editing in rice, with short breeding cycles.

## Introduction

To respond to rapid climate change, it is necessary to improve cultivars of crops and develop rice with resistance to biotic and abiotic stress; this is a primary goal in plant breeding worldwide (Zhang et al., 2018). Recent rapid increases in the global temperature have raised awareness of the fragility of the world’s food supply, and solutions are needed (Siebert et al., 2017). It has recently been established that drought stress is the main cause of the reduction of rice yield, and in fact, drought can have a direct impact on future food shortages (Passioura, 2007; Prabnakorn et al., 2018). In addition, the area of regions subjected to drought stress is expanding, and as drought frequency and intensity increase, drought stress is a problem that must be resolved. In particular, in Asia, which accounts for most of the world’s rice production, annual production of rice suffers a loss of up to 40% due to drought stress (Guo et al., 2021). One specific problem in relation to drought is how to maintain stable yield, while minimizing the drought-related effects of climate change on various food crops. Plants undergo many structural and physiological changes during drought; thus, the breeding of drought stress-resistant crops has become a means by which to strengthen the plants (Yang et al., 2019).

CRISPR/Cas9 became the most powerful and common tool for gene functional analysis and agronomic studies in plant species, especially in rice (Zhou et al., 2017; Hu et al., 2018). This technology is important in studying the agronomic traits and has been widely used for physiological and molecular studies of plants (Tsai and Joung, 2016). In addition, CRISPR/Cas9 is a more precise and higher efficient technique to achieve the goal of improving agricultural traits (Fiaz et al., 2019). Application of CRISPR/Cas9 improved abiotic/biotic stress tolerance and agricultural traits. By genome editing of the flowering suppressor gene *SP5G* (Rehman et al., 2022), rice with promoted flowering time was developed, and rice with improved resistance to biotic stress due to genome-engineering was developed (Zhou et al., 2015). The genome-editing line created by CRISPR/Cas9 proved that YK17S is a superior yield potential gene in *indica* rice (Barman et al., 2019). In addition, CRISPR/Cas9 provides researchers with the opportunity to further research by increasing the understanding of various genes (Liu et al., 2020).

When plants are exposed to drought, they are subjected to many simultaneous negative effects, such as apoptosis, cell aging, reactive oxygen species (ROS) accumulation, and the deterioration of growth development (Chen et al., 2018; Liu et al., 2019; Singh et al., 2019). Recent research has shown that ROS play an important role in plants as signaling factors that transmit in response to major plant growth stimuli, such as stress, pathogen intrusion, and programmed cell death (Qi et al., 2017; Fichman and Mittler, 2020). According to evolutionary theory, photosynthetic life has played an important role in the development of almost all life on Earth due to oxygen release, which is also unfortunately the source of ROS (Cezary et al., 2018). Plants can convert oxygen into ROS in organelles, such as mitochondria and chlorophyll through various common metabolic reactions (Munro and Treberg, 2017). Cellular senescence due to plant ROS accumulation begins with the onset of senescence during the development of all tissues; it decreases photosynthesis, chlorophyll degradation, and protein synthesis ability, leading to the loss of cellular homeostasis and ultimately apoptosis (Huang et al., 2019). In addition to developmental aging, the overall aging process of plants is affected by various biotic and abiotic stresses, including nutrient deficiency, temperature, pathogen attacks, and humidity. Senescence-associated genes respond to drought and cause substantial yield loss (Uji et al., 2017; Jiang et al., 2018). Plants are typically settled in one place; thus, they cannot escape environmental stresses that are unfavorable to their growth. Due to their immobility and exposure to stress, plants have developed numerous defense mechanisms that minimize or eliminate the damage they receive (Ilyas et al., 2021). In particular, plants overcome stress by synthesizing substances, such as proline, raffinose, and glycine betaine under different stress situations including drought; these substances help stabilize cell structure and remove excessively generated ROS including hydrogen peroxide (H_2_O_2_), singlet oxygen, and superoxide anions (Nahar et al., 2018; Verma et al., 2019).

Traditional breeding and transformants have been effectively used to breed stress-tolerant cultivars; indeed, such techniques have facilitated plant growth during drought and solved several drought-related problems (Bai et al., 2018). However, developing cultivars that can improve various stresses resistant and maintain a stable yield requires a deep understanding of plant genetics. In addition, traditional breeding is time-consuming, labor-intensive, and costly. However, novel plant breeding techniques applying CRISPR/Cas9 have been proposed as a new breeding method that can accurately and rapidly improve crop traits (Fiaz et al., 2021a). In previous studies, several quantitative trait loci and genes that induce apoptosis during drought were discovered (Shamloo-Dashtpagerdi et al., 2020; Ganie and Ahammed, 2021); however, validating these results during drought conditions is a complex process. Biotechnology holds the key to solving these problems.

CRISPR/Cas9 is an important genome-editing technology used to create plant varieties with a wide range of traits that solve various problems (Li et al., 2017, 2020). In addition, the application of CRISPR/Cas9 made it possible to understand the functions of various previously unresolved genes (Fiaz et al., 2021b). In the present study, to characterize the functional role of *Oryza sativa SAP* (*OsSAP*) in rice during drought, genome-edited rice plant was developed using CRISPR/Cas9 technology. The morphological and molecular changes in this gene editing plant during drought were compared with the changes in the rice cultivar, *O. sativa* spp. *japonica* cv. Ilmi (Ilmi). Consequently, we found that *OsSAP* induced cellular senescence and apoptosis during drought. Specifically, our results suggest that *OsSAP* accelerates cellular senescence during exposure to drought stress and acts as a negative regulator of cells. Additionally, *OsSAP* was found to function in processes related to apoptosis and cellular senescence during various environmental stressors. Our findings will facilitate further research to elucidate the underlying mechanism of these effects.

## Materials and Methods

### Plant Materials and Development of Genome-Editing Rice

GeD lines were generated using Ilmi as a background. The generated GeD, and Ilmi were cultivated at 28/26°C during a 16/8-h light/dark photoperiod. Three sgRNAs (sgRNA 1–3) were designed using the CRISPR RGEN Tools^1^ program to target the first exon of *OsSAP* (*Oryza sativa Senescence-associated protein*, Accession no. AB734097) and edit the genome (Park et al., 2015); these sgRNAs were integrated into the pRGEB32 vector, which is capable of expressing *Cas9* in plants (Mikami et al., 2015). The obtained binary vector was transformed through calli derived from Ilmi using *Agrobacterium tumefaciens* EHA105 and the rice plant was regenerated as previously described (Yukoh and Toshihiko, 2008). We also used *OsSAP*-T_6_ lines for compared GeD lines related to drought tolerance were acquired from Prof. Ubaidillah (Ubaidillah et al., 2013).

### Detection of Genome-Edited Rice and Assay of Transgene-Free Plant Lines

Genomic DNA was extracted from rice leaves using a DNeasy Plant Mini Kit (QIAGEN, Cat. 69104, Hilden, Germany) according to the manufacturer’s instructions. To amplify the target genome region of CRISPR/Cas9, PCR analysis was performed with primers using these region sequences. The amplified PCR product was purified and mutants for which the target gene was genome-edited were detected through Ilmi sequences and alignment *via* sequencing chromatogram analysis. For all regeneration GeD plants, each line of the T_0_ generation was analyzed for the target region of *OsSAP*.

### RNA Isolation and Gene Expression Analysis

Roots, stems, and leaves were sampled at the seedling stage of rice, whereas the roots, stems, leaves, and panicles at the heading stage were sampled. Total RNA was extracted from the sampled organs using the RNeasy Micro Kit (QIAGEN, Cat. 74004, Hilden, Germany) according to the manufacturer’s instructions. Subsequently, cDNA was synthesized using 1 μg of total RNA from each sample with the qPCRBIO cDNA Synthesis Kit (PCRBIOSYSTEMS, United States), used according to the manufacturer’s instructions. The synthesized cDNA was diluted 1:10 (v/v) using deionized water and then used for qPCR analysis together with the housekeeping gene *OsActin*. qPCR analysis was performed using an Eco™ Real-Time PCR System (Illumina, Cat. EC-900-1001, CA, United States) with 2X Multi-Star OneStep qRT-PCR Master Mix (BIOFACT, Cat. RQ351-50h, Seoul, Korea); relative expression levels were analyzed according to the manufacturer’s instructions. Cycle threshold values for each sample were normalized using *OsActin* as a standard (Ariani et al., 2004). Samples were each analyzed in five independent biological replicates. The list of primer sets used for the analysis is listed in Supplementary Table S1.

### Drought Stress Treatment and Phenotype Evaluation After Stress

GeD lines, Ilmi, and *OsSAP*-T_6_ lines were grown by filling a Wagner pot with soil. Each pot used in the experiment was filled with the same weight of soil, and water was supplied equally to each pot at a height of 5 cm from the soil. Drought conditions were created by not watering plants when they reached the fourth leaf stage; the water supply was stopped until the soil moisture content reached 20%. When the soil moisture content reached 20%, the plants were watered for 7 days and the survival rate was analyzed (Li et al., 2013). To analyze growth, shoot length, root length, chlorophyll content, number of tillers, and 1,000-grain weight, data were collected when the soil moisture content was 20%.

### Detection and Measurement of H_2_O_2_

To detect H_2_O_2_ after drought treatment, the 3,3′-diaminobenzidine (DAB) staining method of Liu et al. (2014) was modified and used. After drought treatment, leaves were cut to a length of 5 cm and then submerged in 1-mg/ml DAB (pH 3.8) to be dyed in darkness at 25°C for 16 h. To prevent the penetration of DAB solution from being disturbed by foreign substances on the leaves, they were cut and their surface was washed with deionized water. After decolorization using 100% ethanol, the reaction of H_2_O_2_ on each leaf was analyzed through newly created brown spots. DAB staining was analyzed on leaves of five different plants for each line and representative images were collected. In addition, to analyze the content of H_2_O_2_ contained in leaves, H_2_O_2_ was extracted using the method proposed by Rao et al. (2000). The content of H_2_O_2_ contained in plant leaves was determined *via* the Quantitative Peroxide Assay Kit (Thermo Fisher, Cat. 23280, Seoul, Korea), used according to the manufacturer’s instructions. Leaf extracts were measured at OD_560_ on a spectrometer. The absorbance values measured for each leaf were calibrated with the standard curve generated with from a known H_2_O_2_ concentration.

### NBT Staining

To evaluate the generation of O_2_^−^ in the leaves of plants after drought stress, 0.1% nitro blue tetrazolium (NBT) solution was added to 10-mM potassium phosphate buffer (pH 7.8), and then leaf samples were cut to a length of 5 cm and precipitated (Fitzgerald et al., 2004). They were incubated in darkness at 25°C for 16 h. To analyze the blue spots generated by the reaction of NBT and O_2_^−^, the chlorophyll contained in the leaves was removed. To remove chlorophyll, the leaves were fixed in alcohol lactophenol (2:1:1, 95% ethanol: lactic acid: phenol) at 65°C for 30 min before they were rinsed with 50% ethanol. Afterward, NBT was found to react with superoxide on the surface of the leaf to form blue spots.

### Analysis of MDA, CAT, POD, SOD, and Proline Content

Proline content was determined using the method from Bates et al. (1973). After drought treatment on GeD, and *OsSAP*-T_6_ lines, 0.5 g of leaf was ground, and homogenized with 3% sulfosalicylic acid (w/v) at 100°C for 10 min. This was then centrifuged at 4,500 RPM for 30 min after 10 min. Subsequently, 2 ml acidninhydrin and 2-ml glacial acetic acid were added to 2 ml of the supernatant, boiled at 100°C for 40 min, and then cooled using ice. Proline levels in the drought-treated samples were determined at OD_520_. MDA content was analyzed *via* the method outlined by Heath and Packer (1968). A 1-g leaf sample was placed in 10 ml of 10% trichloroacetic (v/v) reagent and was homogenized, after which it was centrifuged at 4,500 RPM for 10 min. Next, 2 ml of the supernatant was reacted with 2 ml of thiobarbituric acid at 100°C for 15 min and then cooled with ice before it was analyzed at OD_450_, OD_532_, and OD_600_ to determine MDA concentration. Finally, the MDA content was determined by applying the formula (μmol L^−1^) = 6.45 (OD_532_ − OD_600_) − 0.56 OD_450_, which was proposed by Shi et al. (2012). Catalase (CAT) activity was analyzed by modifying the method of Beers and Sizer (1952). The 3.0 ml reaction mixture contained 2 ml of sodium phosphate buffer (50 mM at pH 7.0), 0.5 ml of H_2_O_2_ (40 mM), and 0.5 ml of enzyme extract. The decomposition of H_2_O_2_ at OD_240_ was analyzed. The activity of superoxide dismutase (SOD), an antioxidant enzyme, under drought stress was analyzed according to the protocol of Duan et al. (2012). The unit of SOD activity was determined as the minimum amount of enzyme required to inhibit the photochemical reduction of NBT chloride by 50% at OD_560_. Peroxidase (POD) activity was analyzed by the guaiacol oxidation method proposed by Weydert and Cullen (2010). The POD reaction mixture (3 ml) contained 50 mM sodium acetate buffer (pH 5.0), 20-mM guaiacol, 40-mM H_2_O_2_, and 0.1-ml enzyme extract. The prepared POD reaction mixture was analyzed at OD_240_ at 1 min intervals; data were recorded before 3 min had passed after the start of the reaction.

### Strategy Establishment for Short Breeding Cycle of CRISPR/Cas9 GE Rice

*Oryza sativa Senescence-associated protein* genome-edited plants with CRISPR/Cas9 were analyzed through genomic sequencing. Regenerated plants from callus were named T_0_ plant, and seeds harvested from T_0_ plant were named T_1_ seed. Also, when T_1_ seeds are sowing and transplanted into the field, they are named T_1_ plants, and the seeds harvested from T_1_ plants become T_2_ seeds. Among genome-edited plants, homozygous types were selected from T_0_. An accession number was assigned to each tiller of the T_0_ plants, and T_1_ seeds were harvested, and each was assigned an accession number. T_1_ seeds were transplanted in one row for each line in the field, and 25 plants were transplanted for each row. Twenty-five plants were planted in each line, and the plants that died after growth were replaced with purple rice and were not used for subsequent analysis. In T_1_ plants, T_2_ seeds were harvested in bulk. In each generation, the expression level of *Cas9* and the target gene, *OsSAP*, was analyzed using *OsActin* as a control.

### Statistical Analysis

All data were statistically analyzed using one-way ANOVA and Duncan’s multiple range tests *via* the SPSS program (IBM SPSS Statistics, version 22, NC, United States). Value of *p* < 0.05 was considered statistically significant.

## Results

### Generation of *OsSAP* Genome-Editing Lines Using CRISPR/Cas9 System

**Figure 1:**
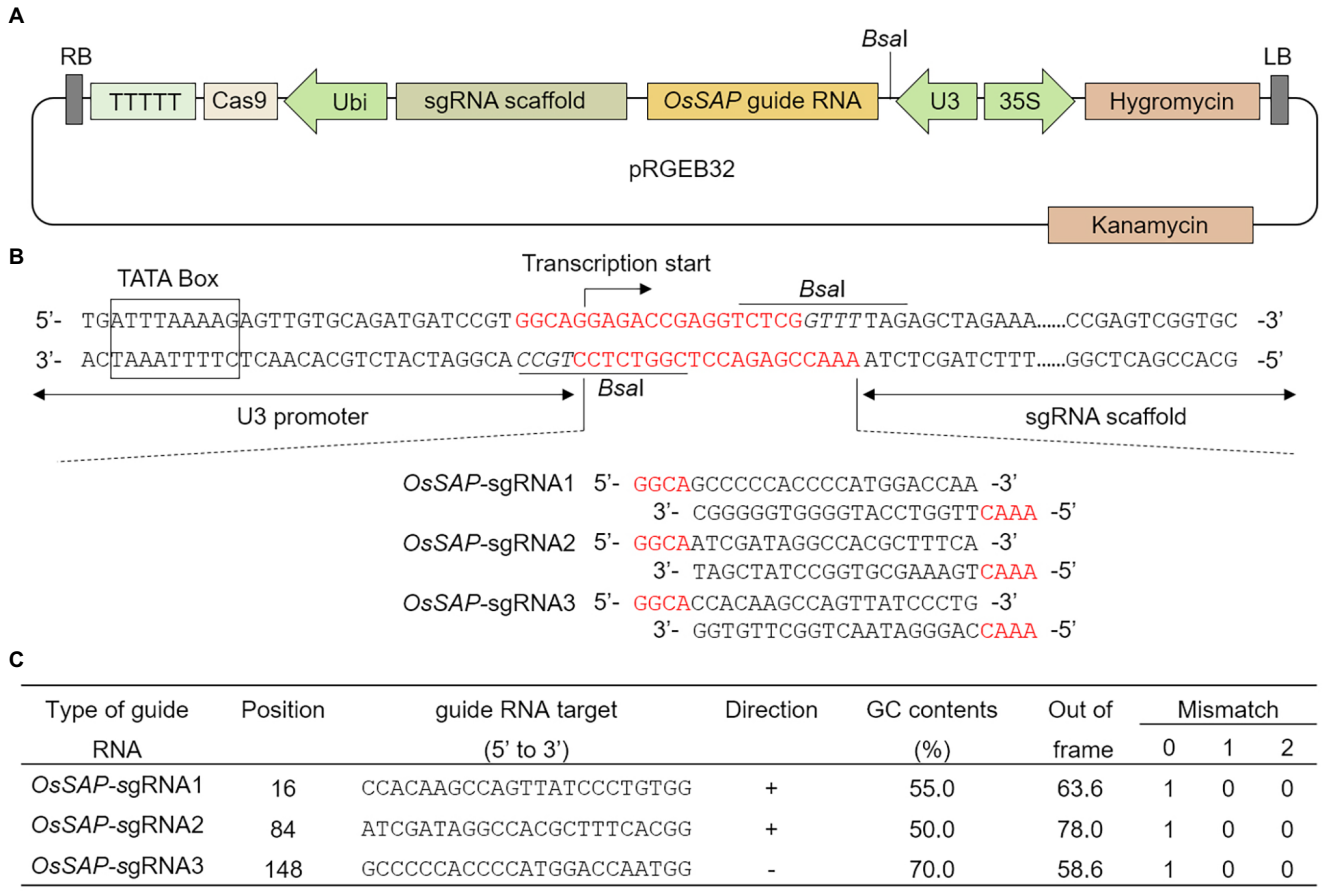
CRISPR/Cas9 vector construct and guide RNA design for *Oryza sativa Senescence-associated protein* (*OsSAP*) genome editing. **(A)** Schematic diagram of the pRGEB32 vector used for genome editing of *OsSAP* through CRISPR/Cas9 expression in rice. The guide RNA and sgRNA scaffold are expressed by the U3 promoter. Cas9 is expressed by the ubiquitin promoter (Ubi). Hygromycin (HPT) is expressed by the CaMV35S promoters (35S). LB and RB: left border and right border, respectively. **(B)** In the CRISPR/Cas9 vector, *OsSAP* guide RNA inserted at the site of the *Bsa*I restriction enzyme site. **(C)** Three sgRNAs were designed for genome editing of *OsSAP*. The GC content of guide RNAs was set to 50–70%. Out-of-frame and mismatch values indicate the possibility that the guide RNA will act off-target. The out-of-frame value was set to 60 or more, and a mismatch of 1-0-0 meant that the designed guide RNA does not have the same sequence as *OsSAP*.

**Figure 2:**
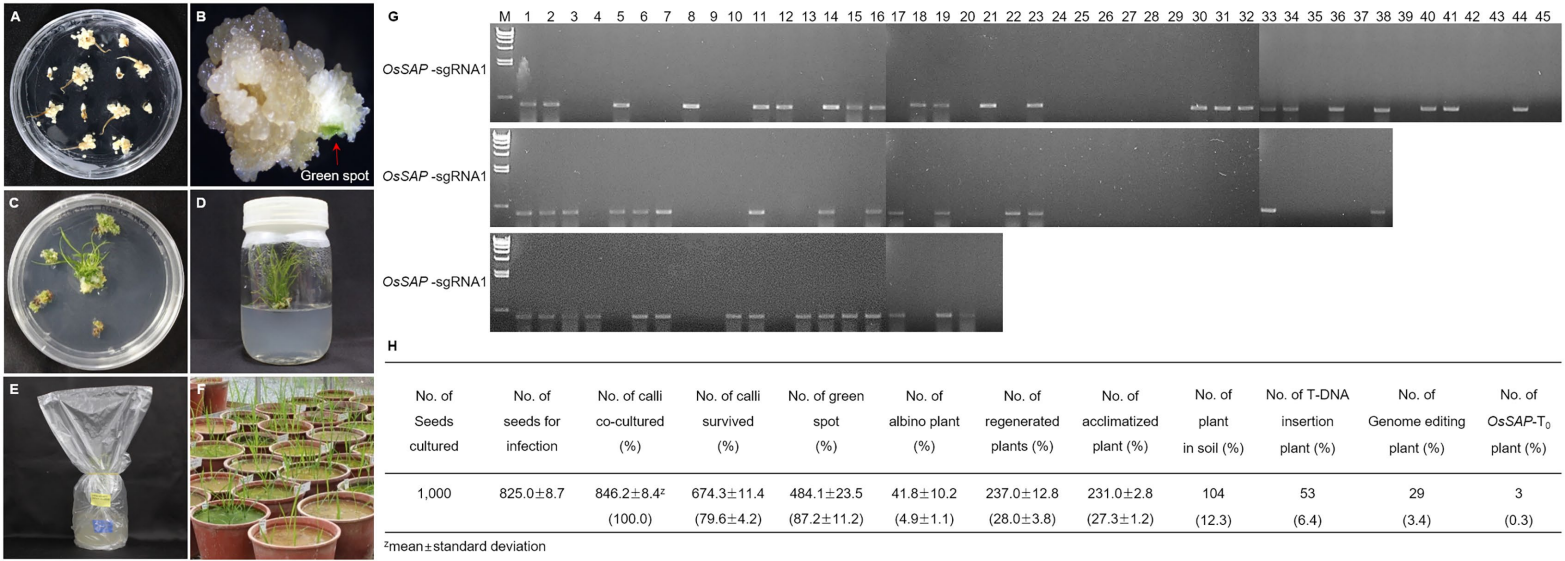
Tissue culture system for development of *OsSAP* genome-editing rice. **(A)** When selecting seeds that induced callus, the seeds were clean and uncontaminated. After 21 days, callus was used for inoculation. **(B)** *Agrobacterium* was used to inoculate the callus and green spots formed in the regeneration media after 7 days. **(C)** Shoots from the green spot and a regenerated plant developed after 45 days in regeneration medium. **(D)** Shooting callus is transferred to new regeneration media for rooting for 30 days. **(E)** To acclimatize the regenerated plants, the lid of the medium was opened and covered with a plastic bag for 3 days. **(F)** After acclimatization, regeneration plants were transplanted into soil. After 90 days the T_1_ seed can be harvested. **(G)** To analyze T-DNA insertion in regenerated plants, PCR amplification was performed using a hygromycin primer set. The hygromycin sequence was amplified in regeneration plants. The amplified products were loaded on 0.8% agarose gel. M: λ/*Hin*d III size marker, Numeric: number of regenerated plants. **(H)** Plant regeneration of the *OsSAP* genome-editing plant.

**Figure 3:**
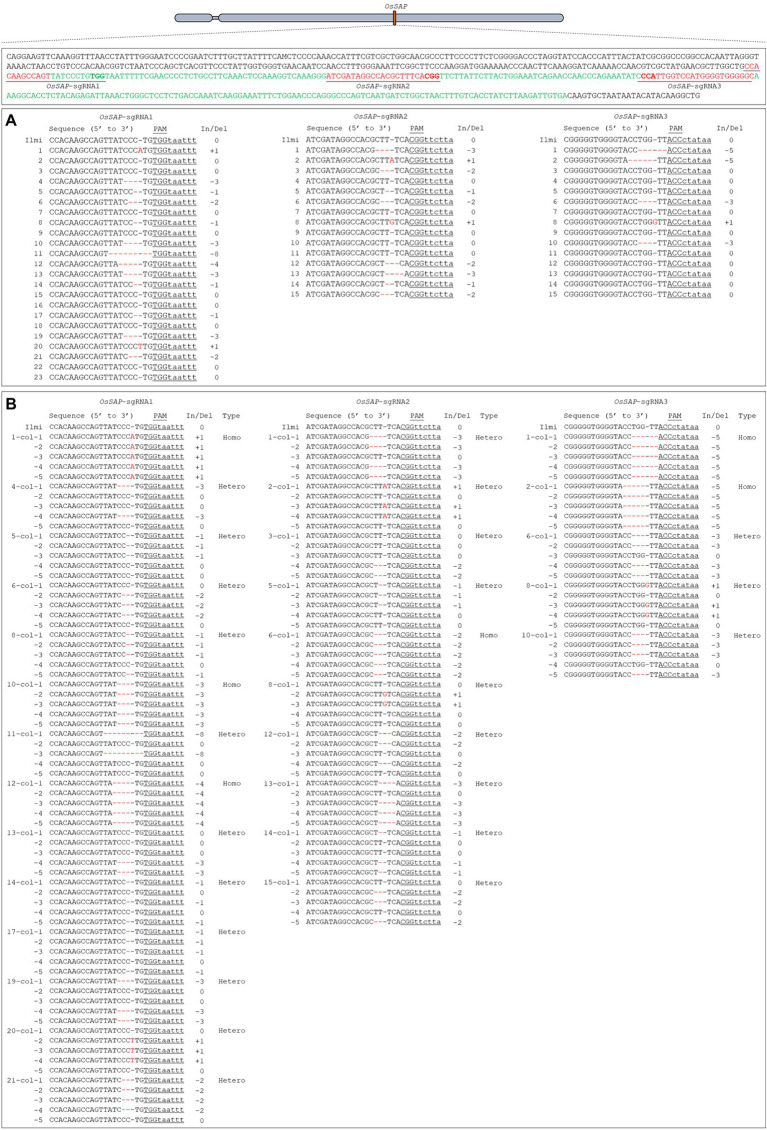
Guide RNA design and genome-editing analysis of target sequence for *OsSAP* genome editing. **(A)** The insertion or deletion sequences of target regions are ranked as hyphens (−) with deletion, or plus (+) with insertion. When *OsSAP*-sgRNA1 was used, 14 genome editing regenerated lines were edited; with *OsSAP*-sgRNA2, 10 were edited, and with *OsSAP*-sgRNA3, five regenerated lines were analyzed. **(B)** Analysis of CRISPR/Cas9-mediated genome-editing type. When guide *OsSAP*-sgRNA1 was used, editing occurred in 14 genome-editing lines, of which three regenerated lines were homozygous and 11 were heterozygote. In *OsSAP*-sgRNA2, out of 10 genome-editing lines, only one was homozygous and the rest were heterozygote. For *OsSAP*-sgRNA3, five genome-editing lines were edited, with two of those lines being homozygous, and the others were heterozygote. Red color: *OsSAP*-sgRNAs sequence. Green color: Motif sequence of *OsSAP*.

To evaluate the role of *OsSAP* in drought conditions, we developed GeD_0_ (Genome-editing Drought gene *OsSAP* 0 generation) lines through genome editing of *OsSAP* using CRISPR/Cas9. In the CRISPR/Cas9 vector, which can be expressed in rice, the *OsSAP* guide RNA is regulated by the U3 promoter (Figure 1A). The guide RNA is inserted into the *Bsa*I site of the pRGEB32 vector (Figure 1B). Three guide RNAs were designed, and the GC contents were set at 50–70% (Figure 1C). Afterward, the CRISPR/Cas9 vector, which the sgRNA was inserted into, was transformed into the callus by *Agrobacterium* medium (Figures 2A–F). For callus inoculation, callus cultured for 21 days were selected (Figure 2A). After co-culture with *Agrobacterium* for 3 days, green spots were investigated after 7 days in the regeneration medium (Figure 2B). After 45 days on the regeneration plate, shoot growth was almost completed (Figure 2C), and culture was performed for rooting on a new regeneration plate for 30 days (Figure 2D). After 3 days of acclimatization (Figure 2E), T_0_ plants were transplanted into pots for T_1_ seed harvesting (Figure 2F). A total of 237 regenerated plants were developed, but only 104 regenerated plants survived when transplanted into soil after acclimatization. Among 104 regenerated plants, there were 45 regenerated plants with genome editing *via OsSAP*-sgRNA1, and T-DNA insertions were analyzed in 23 of them (Figure 2G). There were 38 regenerated *OsSAP*-sgRNA2 plants, and T-DNA insertion was analyzed in 15 of them. When *OsSAP*-sgRNA3 was used, only 15 out of 21 regenerated plants had T-DNA insertion. Around 1,000 Ilmi seeds were used for callus culture, and 237.0 ± 12.8 regenerated plants were developed. Among them, T-DNA was successfully inserted in 53 regenerated plants (Figure 2H). The editing type of *OsSAP* was analyzed with each sgRNA (Figure 3). When *OsSAP*-sgRNA1 was used, deletion occurred in 12 of 23 regenerated plants. There were also two insertions, and nine did not respond to genome editing (Figure 3A). Among the regenerated plants, the editing was homozygous in three plants (Figure 3B). In *OsSAP*-sgRNA2 plants, there were eight deletions, two insertions, and six did not respond to editing. In one of these plants, the homozygous edited type was analyzed. In *OsSAP*-sgRNA3 plants, there were four deletions, one insertion, and 10 were not edited. Among editing plants, two plants were edited homozygous. For continuous research, three plants (designated as GeD_0_ 1–1, GeD_0_ 3–1, and GeD_0_ 3–2) were selected for the generation of GeD lines.

### CRISPR/Cas9-Mediated *OsSAP* Genome-Editing Lines Exhibited Reduced Drought Tolerance

To evaluate the response of *OsSAP* to drought conditions, the soil moisture content of the GeD, and *OsSAP*-T_6_ lines was reduced to 20%. At this level, the survival rate, shoot length, root length, chlorophyll content, number of tillers, and 1,000-grain weight of each line were investigated (Supplementary Figure S1). These variables showed no significant differences in all lines of GeD, and *OsSAP*-T_6_. The survival rates of GeD lines (GeD_0_ 1–1, GeD_0_ 3–1, and GeD_0_ 3–2) under drought stress were lower than that of *OsSAP*-T_6_ (43.5% vs. 17.4, 16.8, and 15.6%, respectively). The overall shoot length showed a trend under drought stress: GeD lines (GeD_0_ 1–1, GeD_0_ 3–1, and GeD_0_ 3–2) had shoot lengths of 6.4, 5.8, and 6.1 cm, respectively, which were lower than that of *OsSAP*-T_6_ (15.1 cm). Under drought stress, the root lengths (2.8 cm, 3.5 cm, 4.9 cm, respectively) of GeD lines (GeD_0_ 1–1, GeD_0_ 3–1, and GeD_0_ 3–2) were shorter than or equal to that of *OsSAP*-T_6_ lines (9.1 cm). The chlorophyll content of *OsSAP*-T_6_ plants under drought conditions was 42.1 SPAD; that of the GeD lines (GeD_0_ 1–1, GeD_0_ 3–1, and GeD_0_ 3–2) was lower than that of the *OsSAP*-T_6_ Lines. Drought stress treatment reduced the number of tillers; the tiller number of *OsSAP*-T_6_, 12.3, was larger than that of the GeD lines at (GeD_0_ 1–1, GeD_0_ 3–1, and GeD_0_ 3–2), 2.3, 2.3, and 2.0, respectively. Finally, when 1,000-grain weights were measured in each line after drought stress, the weight of *OsSAP*-T_6_ lines was 20.3 g, whereas the weights of the GeD lines (GeD_0_ 1–1, GeD_0_ 3–1, and GeD_0_ 3–2) were lower at 9.5, 7.8, and 7.1 g, respectively.

### Increasing the ROS Damage in *OsSAP* Genome-Editing Lines

**Figure 4:**
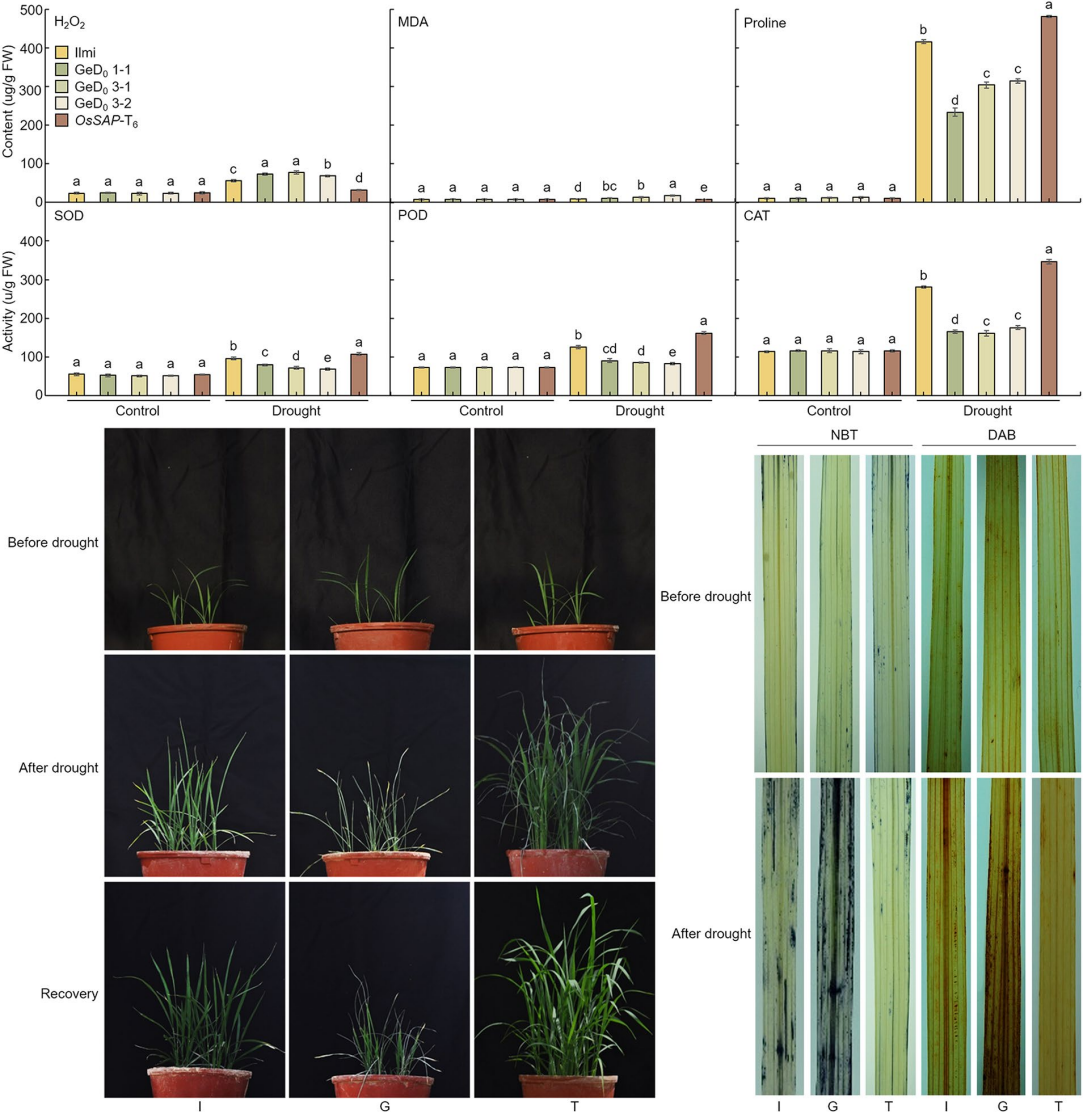
Effects of *OsSAP* on cellular antioxidants and reactive oxygen species (ROS) levels in Ilmi, GeD, and *OsSAP*-T_6_ lines under normal and drought conditions. Under drought conditions, the effects of *OsSAP* on the level of endogenous hydrogen peroxide (H_2_O_2_), the accumulation of malondialdehyde (MDA), the accumulation of proline (proline), the activity of superoxide dismutase (SOD), the activity of peroxidase (POD), and the activity of catalase (CAT). Data presented are expressed as mean ± SD from five independent biological experiments per line. Bars represent means ± SE. Means denoted by the same letter are not significantly different (*p* < 0.05) as evaluated by Duncan’s multiple range test. The GeD lines showed reduced drought tolerance, whereas the *OsSAP*-T_6_ lines showed increased drought tolerance before drought stress, and 20 days after germination. Drought stress treatment; phenotypic changes at 20% soil moisture content. Phenotypes after 7 days of recovery achieved by watering (recovery from 20% soil moisture content). Phenotypes of 3,3-diaminobenzidine (DAB) and nitro blue tetrazolium (NBT) staining when the soil moisture content was 20%. After drought stress treatment, H_2_O_2_ was visualized as brown spots by DAB, and O_2_^−^ as blue spots by NBT. The picture presented is the most representative of five independent biological experiments per each line. I: Ilmi. G: GeD_0_ 1–1. T: *OsSAP*-T_6_. Different letters on columns represent significant (*p* < 0.05) difference between rice lines based on Duncan’s test.

Superoxide dismutase and CAT are important enzymes that protect plant cells from oxidative damage caused by ROS: SOD acts as an antioxidant defense mechanism in almost all living cells by catalyzing the conversion of superoxide (O_2_^−^) to O_2_ and endogenous H_2_O_2_; CAT then catalyzes the conversion of H_2_O_2_ to H_2_O and O_2_^−^. To analyze the ROS scavenging capacity of *OsSAP* under drought stress, GeD, and *OsSAP*-T_6_ lines were subjected to drought stress until the soil moisture content reached 20%. Under control conditions, the H_2_O_2_ contents of GeD, and *OsSAP*-T_6_ lines were the same (Figure 4). However, under the drought stress conditions, the amount of H_2_O_2_ increased and the H_2_O_2_ increase rate differed in each line. In *OsSAP*-T_6_, H_2_O_2_ increased by 31.6 ± 0.9% due to drought stress. However, in the GeD, H_2_O_2_ increased by an average of 71.6 ± 4.5%. Under drought stress, the GeD lines generated more H_2_O_2_ than *OsSAP*-T_6_. All lines of GeD, and *OsSAP*-T_6_ had similar levels of malondialdehyde (MDA) content under control conditions. However, when drought stress was applied, MDA content increased in all analyzed lines relative to the control conditions. However, the *OsSAP*-T_6_ lines increased by only 6.5 ± 0.1% on average, whereas the GeD lines showed an average increase of 12.6 ± 3.2%. Proline production by drought treatment was also different in the GeD lines, compared to the *OsSAP*-T_6_ lines. Under control conditions, almost no proline was generated in all analyzed lines. However, when drought stress was applied, proline content increased overall in all analyzed lines: the average increase in the *OsSAP*-T_6_ line was 494.7 ± 9.1%, which represented an increase of 493.7% ± 9.1%. The average proline content of GeD lines was 283.9 ± 43.9%, which was an increase of 282.9 ± 43.9% when compared with the control. Thus, proline content increased more in the *OsSAP*-T_6_ lines following drought stress application. These findings suggest that the expression of *OsSAP* reduces cell damage in leaves when a rice plant in under drought stress conditions. Under normal conditions, the level of SOD activity was consistent in all lines. However, when drought stress was applied, SOD activity increased in all lines: the SOD activity was highest in the *OsSAP*-T_6_ lines and lowest in the GeD lines. POD activity was also the same in all analyzed lines under normal conditions; however, when drought stress was applied, POD activity was highest in the *OsSAP*-T_6_ lines and lowest in the GeD lines. CAT showed a similar trend to that of SOD: it was highest in *OsSAP*-T_6_ lines and lowest in GeD lines when drought stress was applied. These results suggest the possibility that *OsSAP* increases drought resistance by acting on the oxidative stress response.

When the rice reached the four-leaf stage, the GeD and *OsSAP*-T_6_ lines were subjected to drought stress by stopping the water supply until the soil moisture content reached 20%. Before drought stress treatment, there was no difference in the phenotype of all lines and, the growth rate was similar (Figure 4). However, when water was supplied after drought stress, the recovery of the GeD lines was slow, whereas the recovery of the *OsSAP*-T_6_ lines was rapid. To determine whether ROS production was related to *OsSAP*-mediated resistance to drought stress (with moisture content at 20%, as described earlier), the H_2_O_2_ content of GeD, and *OsSAP*-T_6_ lines after drought stress treatment were analyzed using DAB staining. In the controls, all analyzed lines showed very little staining. Under drought stress, *OsSAP*-T_6_ lines were barely stained but the GeD lines produces many dark and brown spots were on their leaves; indeed, most of their leaves were covered with brown spots. In addition, NBT staining was analyzed to evaluate the relationship between *OsSAP* and O_2_^−^ production under drought conditions. Under control conditions, almost no blue spots were observed in all lines; however, after drought stress, the *OsSAP*-T_6_ lines still showed a similar pattern to the control, whereas the incidence of blue spots increased in GeD lines compared to both. NBT and DAB staining indicated that drought stress generated H_2_O_2_ and O_2_^−^ at increased levels in the GeD lines relative to the levels in the *OsSAP*-T_6_ lines, suggesting that *OsSAP* could control overproduction and regulation of H_2_O_2_ under drought stress. Thus, the expression of *OsSAP* could reduce peroxidation and ROS accumulation by enhancing the activity of major antioxidant enzymes under drought stress conditions.

### *OsSAP* Is Involved in the Expression of Major Stress-Related Regulatory Genes During Drought Stress

**Figure 5:**
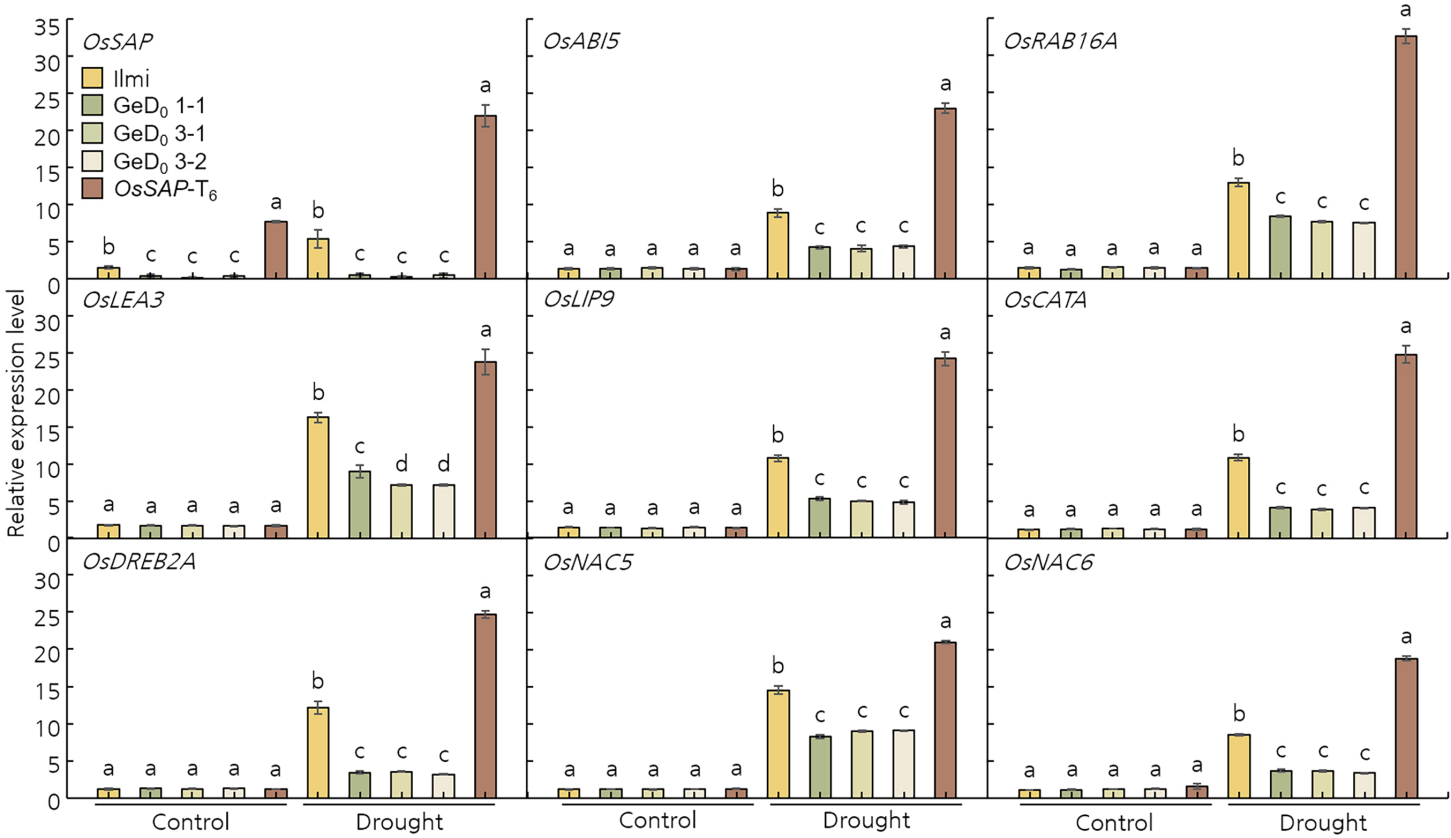
*Oryza sativa Senescence-associated protein* is involved in the expression levels of several critical stress-related transcription factors under drought conditions. Under normal conditions *OsABI5*, *OsRAB16A*, *OsLEA3*, *OsLIP9*, *OsCATA*, *OsDREB2A*, *OsNAC5*, and *OsNAC6* expression levels were the same. However, overexpression of *OsSAP* under drought conditions improved the expression levels of the evaluated stress-related transcription factors. Bars represent mean ± SE. Means denoted by the same letter are not significantly different (*p* < 0.05) as evaluated by Duncan’s multiple range test. Different letters on columns represent significant (*p* < 0.05) difference between rice lines based on Duncan’s test.

To understand the molecular mechanism of *OsSAP* in drought stress, the gene expression level of transcription factors that respond to drought stresses was analyzed. Specifically, after drought stress on GeD, and *OsSAP*-T_6_ lines, relative expression levels of the stress-related transcription factors *OsABI5*, *OsRAB16A*, *OsLEA3*, *OsLIP9*, *OsCATA*, *OsDREB2A*, *OsNAC5*, and *OsNAC6* were examined using qRT-PCR (Figure 5). All analyzed stress-related transcription factors were maintained at low levels or were significantly downregulated in all analyzed lines before drought stress treatment. However, after drought stress, all expression levels were upregulated. Expression levels of these stress-related transcription factors showed contrasting trends in *OsSAP*-T_6_ and GeD lines; significantly lower expression levels were observed in GeD compared to *OsSAP*-T_6_ lines. These results suggest that overexpression of *OsSAP* supports an increase in drought stress tolerance and enhances the expression of stress-related genes.

### Application of Short Breeding Strategy With CRISPR/Cas9-Mediated Genome-Editing Rice

**Figure 6:**
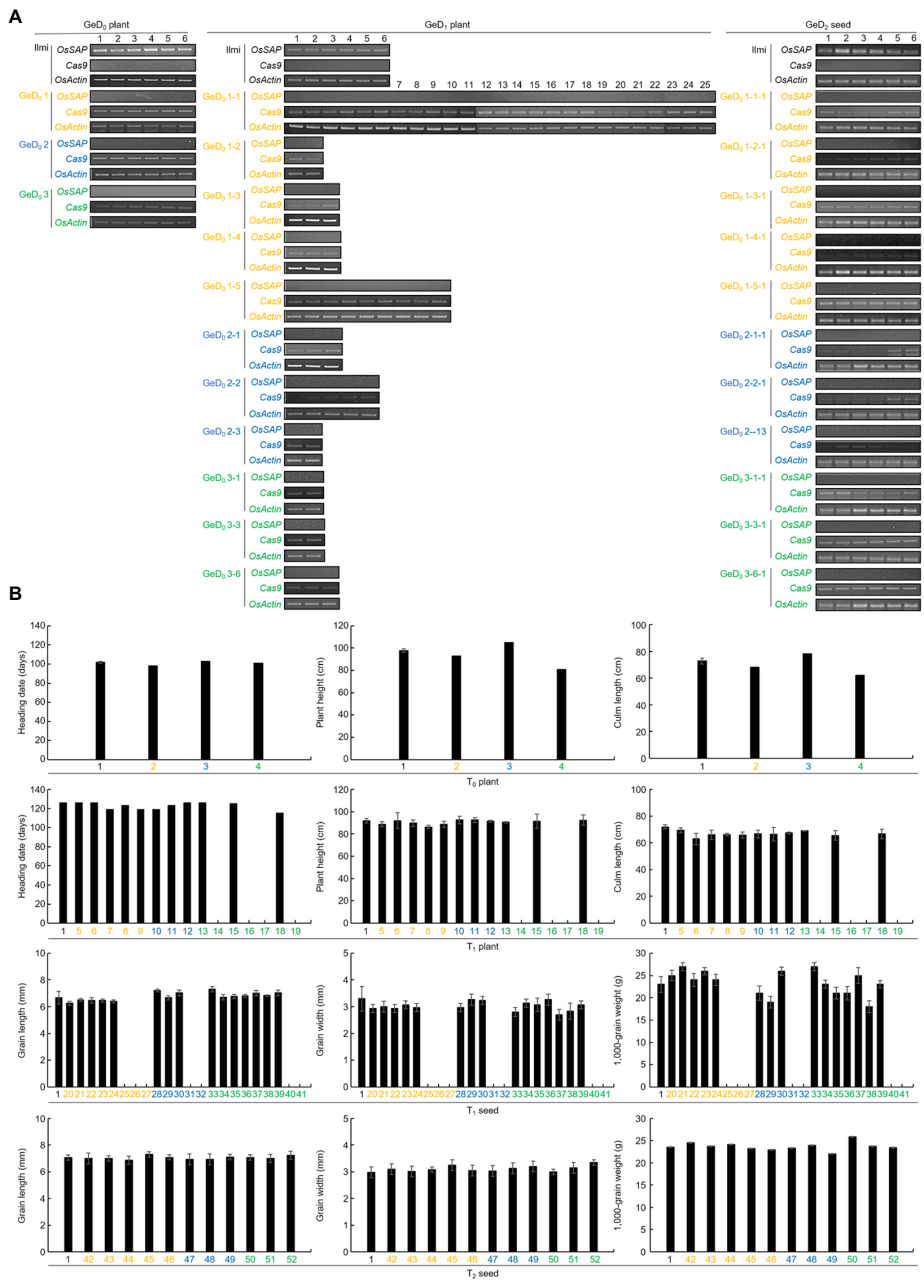
Analysis of the expression level of *OsSAP* and *Cas9* in CRISPR/Cas9-mediated genome-editing lines in each generation through RT-PCR and analysis of agricultural traits of plants and seeds in each generation. **(A)** Expression levels of *OsSAP* and *Cas9* were analyzed for each line in GeD_0_ plants, GeD_1_ plants, and GeD_2_ seeds, with *OsActin* used as a control. In the GeD_0_ plant, Cas9 expression was very strong in all lines with no expression of *OsSAP*. In GeD_1_ plants, *OsSAP* was not expressed. However, *Cas9* was still strongly expressed, or the expression was weakened. In GeD_2_ seeds, *OsSAP* was not expressed, and *Cas9* expression was weakened or maintained. **(B)** Agricultural traits of plants and seeds in each generation of genome-edited plants with CRISPR/Cas9. Since T_0_ plants are each different lines, there is no repeated experiment. T_1_ plants which did not germinate or died early in their growth stage were not investigated. T_1_ seed numbers were assigned to the tiller of the T_0_ plants. Effective tillering was investigated by harvesting the grains, but an accession number was only assigned to the non-productive tiller because there were no grains. For T_2_ seeds, grain length, grain width, and 1,000-grain weights were investigated. The 1 on the x-axis is Ilmi. Yellow color: Genome-editing lines using *OsSAP*-sgRNA1. Blue color: Genome-editing lines using *OsSAP*-sgRNA2. Green color: Genome-editing lines using *OsSAP*-sgRNA3.

Expression levels of *OsSAP* and *Cas9* were analyzed in GeD_0_ and GeD_1_ plants (Figure 6). In all GeD_0_ plants, *Cas9* was expressed at a level similar to *OsActin*, but *OsSAP* was not expressed. In all GeD_1_ plants, *OsSAP* was not expressed; however, *Cas9* was expressed in various concentrations throughout the GeD_1_ lines. *Cas9* was very weakly expressed in GeD_0_ 1–2, GeD_0_ 1–3, and GeD_0_ 1–4, and GeD_0_ 1–1 and GeD_0_ 1–5. *OsSAP* was not expressed in all *OsSAP*-G-2 lines; however, *Cas9* expression of each GeD_0_ 2 line was different. In GeD_0_ 2–1, *Cas9* was expressed very weakly compared to *OsActin*, and in GeD_0_ 2–3, *Cas9* was expressed at a level similar to that of *OsActin*. In addition, GeD_0_ 2–2 showed weaker *Cas9* expression than *OsActin*, but stronger *Cas9* expression than GeD_0_ 2–1. *OsSAP* was not expressed in GeD_0_ 3 lines, and the *Cas9* expression level of all GeD_0_ 3 lines was weaker than that of *OsActin*. The expression levels of *OsSAP* and *Cas9* in GeD_2_ seeds were also analyzed. *OsSAP* was not expressed in all lines of GeD_2_ seeds. In the GeD_0_ 1-1-1, GeD_0_ 1-2-1, and GeD_0_ 1-4-1 lines, *Cas9* expression was weaker than that of *OsActin*. *Cas9* was strongly expressed in GeD_0_ 1-4-1, and *Cas9* expression of GeD_0_ 1-3-1 and GeD_0_ 1-5-1 is similar to that of *OsActin*. In all the GeD_0_ 2 lines, the *Cas9* expression level was lower than that of *OsActin*. In particular, *Cas9* was not expressed in GeD_0_ 2-3-1. In GeD_0_ 3-1-1, the *Cas9* expression level was lower than that of *OsActin*, but the *Cas9* expression level of GeD_0_ 3-3-1 and GeD_0_ 3-6-1 was similar to that of *OsActin*. Agricultural traits of T_0_ plants, T_1_ plants, T_1_ seeds, and T_2_ seeds were investigated. The coefficient of variation (CV) of the heading date, plant height, and culm length of T_0_ plant were 2.5, 12.9, and 11.7%, respectively (Supplementary Figure S2). The CVs of the heading date, plant height, and culm length of T_1_ plant were 3.1, 2.2, and 2.6%, respectively. The CVs of grain length, grain width, and 1,000-grain weight of T_1_ seed were 4.5, 5.6, and 17.1%, respectively, and the CV of grain length, grain width, and 1,000-grain weight of T_2_ seed were 1.9, 3.4, and 4.1%, respectively.

## Discussion

Globally, unpredictable climate change has seriously threatened crop yields due to adverse biotic and abiotic conditions causing major harvest losses worldwide. The International Food Policy Research Institute predicts that rice demand will increase by 30% by 2050, and that drought stress will reduce rice yield by about 13%. CRISPR/Cas9 has developed rapidly in recent years and is now being applied to gene functional analysis and molecular breeding to combat future losses. Rice has adapted to various environments around the world, and as a result, it has become a very important staple food crop worldwide. Recently, however, the demand for new cultivar with improved resistance to abiotic/biotic stressors has increased and will continue to increase as climate change worsens over time. In this study, CRISPR/Cas9 was used to analyze gene function and to apply it to short breeding in the field. Research shows that ROS plays a key role in regulating the development and stress tolerance response of major crops in an era of unpredictable climate change (Moradi and Ismail, 2007; Steffens, 2014). Senescence-associated genes regulated by biotic stress play a key role in the production of ROS, and the sensitivity and function of stress responses vary according to plant types (Kajimura et al., 2010). Senescence-associated genes are reportedly involved not only in plant growth and development but also in plant resistance to various oxidative stresses (Li et al., 2021). *Arabidopsis thaliana AtSOP1* and *SAG29* (Sakuraba et al., 2017) act on salt-stress resistance, whereas *OsDOF24* from rice is involved in the jasmonate-mediated pathway (Shim et al., 2019), and *OsSRLK* is involved in phytohormone-mediated chlorophyll degradation (Ying et al., 2020). These genes play an important role in conferring resistance to various biotic/abiotic stresses and establishing a complex signal transduction network in the aging and growth processes of plants.

We found that *OsSAP* genome editing make more susceptible against drought stress while decrease survival rate, shoot and root lengths, chlorophyll content, tiller number, and 1,000-grain weight. Previous studies have reported that introducing external genes and genome editing into rice and crops can negatively affect plant growth, development, and yield due to signal disturbance of internal genes (Baxter et al., 2015; Metje-Sprink et al., 2020). However, in our study, tiller number and 1,000-grain weight (which are major factors of quantity) were the same after genome editing of *OsSAP* under normal conditions as they were in Ilmi, that is, no effects were observed. However, under drought stress, *OsSAP* conferred drought tolerance by positively affecting these variables. Drought stress promoted the generation of excessive ROS in rice. H_2_O_2_ and O^2−^are both representative ROS caused by drought stress (Jira-Anunkul and Pattanagul, 2021; Mohammad et al., 2021). When ROS is generated in high amounts due to various stressors, antioxidant enzymes specifically act on each active oxygen to remove it (Dvořák et al., 2021). MDA, for example, inhibits cell function and induces cell membrane degradation while also acting as an important mediator in ROS scavenging (Sun et al., 2020). Often, the level of MDA in cells can be used to predict the occurrence of programmed cell death and analyze plant cytotoxicity (Wang et al., 2017). SOD specifically removes O^2−^, whereas CAT and POD are representative antioxidant enzymes that specifically remove H_2_O_2_ (Zhang et al., 2019). High levels of H_2_O_2_ were detected in the GeD lines, and numerous spots were formed as a result of DAB and NBT staining; this indicates that excessive H_2_O_2_ and O^2−^ were present. However, in the *OsSAP*-T_6_ lines, H_2_O_2_ and O^2−^ were hardly detected. The *OsSAP*-T_6_ lines also showed an increased activity of antioxidant enzymes, that is, CAT, SOD, and POD, whereas the GeD lines maintained antioxidant enzyme activity at a relatively low level. Therefore, *OsSAP* seems to improve drought tolerance by protecting against the oxidative damage caused by stress, which is achieved by improving the ability of plants to remove ROS when faced with drought stress (Qi et al., 2018). Plants have developed and evolved a complex antioxidant system to resist stress from various free radicals; consequently, they can maintain high expression levels of antioxidant enzymes, such as CAT, SOD, and POD, to confer stress resistance (Huang et al., 2019). We monitored the expression levels of various transcription factors related to drought stress, such as *OsABI5* (Skubacz et al., 2016), *OsRAB16A* (Ganguly et al., 2012), *OsLEA3* (Xiao et al., 2007), *OsLIP9* (Chen et al., 2015), *OsCATA* (Yong et al., 2017), *OsDREB2A* (Agarwal et al., 2007), *OsNAC5* (Song et al., 2011), and *OsNAC6* (Lee et al., 2017), to understand molecular stress tolerance to *OsSAP* under drought conditions. The selected genes are known to be involved in resistance to abiotic stresses, such as drought stress, in various plants. These genes were downregulated in the GeD lines when compared with their expression in Ilmi under drought stress, which is consistent with the level of activity of ROS antioxidant enzymes observed. We found that overexpression of *OsSAP* improved the ability of plants to remove ROS and increased the transcript levels of genes required to resist drought stress. In addition, *OsABI5*, *OsRAB16A*, *OsLEA3*, *OsLIP9*, *OsCATA*, *OsDREB2A*, *OsNAC5*, and *OsNAC6* are genes that improve the tolerance of various plants to abiotic stress. Senescence-associated genes belong to a large family with functions related to cell death under various abiotic/biotic stresses (Pan et al., 2016).

**Figure 7:**
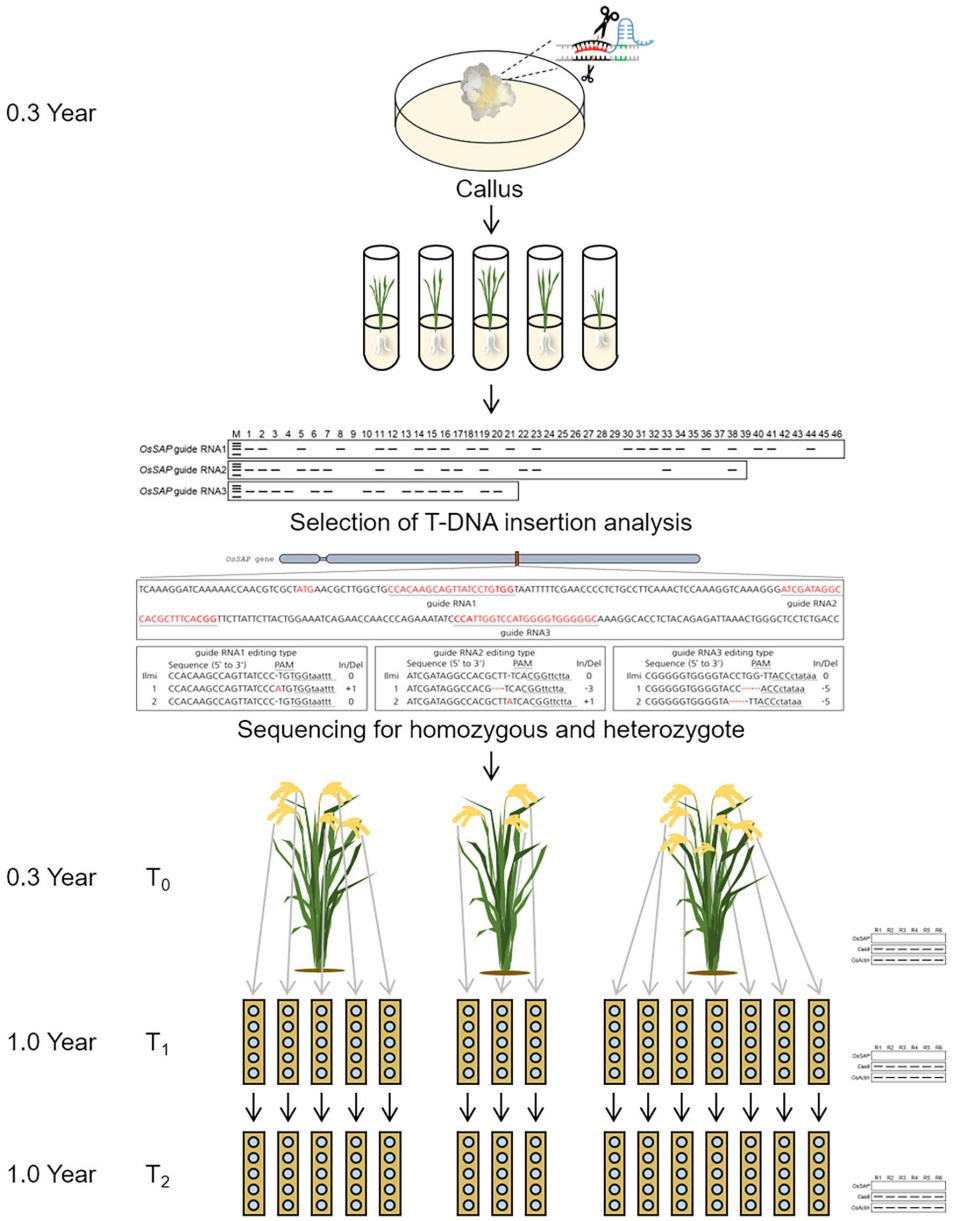
Scheme of short breeding cycle using CRISPR/Cas9 in rice. *Agrobacterium*-mediated transformation used for genome editing with CRISPR/Cas9. In regenerated plants, a gene was selected and edited by T-DNA insertion and sequencing. On the T_0_ plant, each spike is assigned a different accession number. T_1_ plants are transplanted to one row for each line. T_2_ seeds are harvested in bulk.

Using these research results, we propose a response model for *OsSAP* under drought stress conditions (Supplementary Figure S3). Drought stress induces rapid overexpression of *OsSAP*, which in turn induces the expression of antioxidant enzymes, such as CAT, SOD, and POD, which increases the overall activity of these enzymes. Therefore, the scavenging ability of ROS caused by drought stress is improved, and drought tolerance emerges. *OsSAP* also increases drought tolerance by enhancing the expression of stress-related transcription factors. CRISPR/Cas9 can shorten the breeding period in rice (Figure 7). The CRISPR/Cas9 vector transformed into the callus by the *Agrobacterium*-mediated method; T_0_ plants were selected through T-DNA insertions and target gene editing sequences. Proper T_0_ plant selection and subsequent harvest of a T_1_ seed from a T_0_ plant takes 0.3 years. Here, we used the “ear-to-row method” to apply CRISPR/Cas9 to rice breeding. In this study, GeD_0_ 1 had five spikes, GeD_0_ 2 had three spikes, and GeD_0_ 3 had seven spikes. In the field, T_1_ plants were transplanted in one row for each spike, and T_2_ seeds were harvested in bulk. Agricultural traits of the T_0_ plants, T_1_ plants, T_1_ seeds, and T_2_ seeds were investigated, and the CV of both T_1_ plants and T_2_ seeds were lower than 5%. Taken together, our results suggest that CRISPR/Cas9 can accurately genome edit homozygous target genes in T_0_ plants, and short breeding cycles are possible evidenced by the low CV in T_1_ plants and T_2_ seeds. Therefore, CRISPR/Cas9 can be used as a simple, rapid, and powerful tool in crop improvement, and will greatly advance molecular design in crop breeding. Our results suggest that a high efficiency of CRISPR/Cas9 genome editing and breeding in rice is possible, as a significant percentage of our T_0_ plants were homozygous for the target edited gene; however, not all target sequences were edited in T_0_ plants as evidenced by an abundance of heterozygotes and unedited genes.

In this research, overexpression of *OsSAP* during drought stress increased drought resistance, whereas genome editing targeted at *OsSAP* increased drought susceptibility. Therefore, these findings provide evidence that *OsSAP* could be a potential candidate gene for the improvement of rice growth under drought stress. This would, in turn, improve the major agricultural traits of plants in stressed conditions, leaving them fairing as well as or better as plants in “normal” conditions (Sade et al., 2018; Piao et al., 2019). To resist severe abiotic/biotic stressors, cultivars are being developed with improved stress tolerance through the overexpression of stress-related genes and *via* genome editing. However, the continuous overexpression of such genes can induce the development of malformed plants and ultimately reduce their yield (Baxter et al., 2015). Thus, even if drought resistance is improved, the high proportion of malformed plants and lower yields would prevent their usage in farms. However, with overexpression of *OsSAP*, the characteristics of major agricultural traits, including Ilmi and yield amount, were the same as those found under normal conditions. Under drought conditions, when *OsSAP* was overexpressed, the major agricultural traits, including yield, were improved relative to those of Ilmi. In the era of unpredictable climate change, our research into *OsSAP* provides insights that could help resolve food shortages and food security, worldwide.

## Conclusion

By applying CRISPR/Cas9, the functional loss of *OsSAP* interfered with the antioxidant defense by reducing the activation of SOD, POD, and CAT, and finally, the negative effects of various agricultural traits due to the accumulation of ROS and MDA resulted in leaf aging and reduction 1,000-grain weight. These results suggest that *OsSAP* plays an important role in inducing tolerance to drought stress, is related to leaf senescence, and can be used as a potential gene for high yield in drought stress when breeding new rice cultivars. Our research demonstrated promising applications of CRISPR/Cas9-mediated genomic engineering for basic and applied studies of rice and various plants in preparation for future unpredictable climate change. When a genome-edited rice plant is developed by CRISPR/Cas9 technology, homozygote type can be developed in T_0_ plants. In addition, the expression of the target gene was continuously removed from the T_0_ plant, and the expression of *Cas9* remained stable or weakened as the course of generation advancement progressed. Although the expression of Cas9 was reduced, the expression of the target gene was still not analyzed. If CRISPR/Cas9 is applied to breeding, the desired trait can be improved by editing the target gene in early generation with homozygote type. Finally, CRISPR/Cas9 enables a short breeding cycle.

## Data Availability Statement

The datasets presented in this study can be found in online repositories. The names of the repository/repositories and accession number(s) can be found in the article/Supplementary Material.

## Author Contributions

J-RP and E-GK: conceptualization of experiments, methodology and research plan, and writing the original draft. Y-HJ: formal analysis. E-GK and Y-HJ: conducting experiments. RJ, MF, and MU: participating in phenotype evaluations. J-RP: reviewing and editing the writing. K-MK: project administration. All authors contributed to the article and approved the submitted version.

## Funding

This work was supported by a grant from the New Breeding Technologies Development Program (Project No. PJ016531012022), Rural Development Administration, Republic of Korea. This work was supported by the National Research Foundation of Korea Grant funded by the Korean Government (NRF-2021M3E5E6022715).

## Conflict of Interest

The authors declare that the research was conducted in the absence of any commercial or financial relationships that could be construed as a potential conflict of interest.

## Publisher’s Note

All claims expressed in this article are solely those of the authors and do not necessarily represent those of their affiliated organizations, or those of the publisher, the editors and the reviewers. Any product that may be evaluated in this article, or claim that may be made by its manufacturer, is not guaranteed or endorsed by the publisher.

## Footnotes

^1^ http://www.rgenome.net/
