# Supplementary material for: Applications of CRISPR/Cas9 as New Strategies for Short Breeding to Drought Gene in Rice
Source: Front Plant Sci. 2022 Feb 24;13:850441. doi: 10.3389/fpls.2022.850441 (PMC8908215; doi:10.3389/fpls.2022.850441)
Supplement: Supplementary file 1 [file Data_Sheet_1.docx]

# SUPPLEMENTARY MATERIAL

**Supplementary Table S1 ǀ** Primer set used for qRT-PCR, construction of overexpression and genome-editing rice, and for identification of genome editing lines

| Primer Name | Forward/Reverse | Sequence (5’ to 3’) |
| --- | --- | --- |
| *OsActin*-qPCR | Forward | ACCACAGGTATTGTGTTGGACTC |
|  | Reverse | AGAGCATATCCTTCATAGATGGG |
| *OsSAP*-qPCR | Forward | CTGCCTTCAAACTCCAAAGG |
|  | Reverse | CTGGGTTGGTTCTGATTTCC |
| *OsABI5*-qPCR | Forward | AGCGGTGAACCAGTTTGATT |
|  | Reverse | ATCTGCCTGTTTCCTCTCCA |
| *OsRAB16A*-qPCR | Forward | TCAAGTTGAAGGCTGCAATG |
|  | Reverse | AGGTTCACAGACTCGGTGCT |
| *OsLEA3*-qPCR | Forward | TCACTTCAAATTCGGTGCAA |
|  | Reverse | CGACCACCACTTCATACAGC |
| *OsLIP9*-qPCR | Forward | CGTGTCAAGATTGGTCAAGG |
|  | Reverse | TACCCCACACGAAACACAAA |
| *OsCATA*-qPCR | Forward | GCCGGATAGACAGGAGAGGT |
|  | Reverse | TCTTCACATGCTTGGCTTCA |
| *OsDREB2A*-qPCR | Forward | GGCTGAGATCCGTGAACCAA |
|  | Reverse | GGACCATACATTGCCCTTGC |
| *OsNAC5*-qPCR | Forward | CACTGTCAGGTCGATCGTGT |
|  | Reverse | CGATCGAGCACGGTTAATTT |
| *OsNAC6*-qPCR | Forward | GCCGGCGTTCCCGGACCTGGCGGCG |
|  | Reverse | CCGCCGCCGCCGAGGCCGCCGAGGC |
| *OsSAP*-OX | Forward | CACCATGAACGCTTGGCTGC |
|  | Reverse | CATCTCACAATCTTAAGATAGGTGAC |
| *OsSAP*-GE-1 | Forward | GGCAGCCCCCACCCCATGGACCAA |
|  | Reverse | AAACTTGGTCCATGGGGTGGGGGC |
| *OsSAP*-GE-2 | Forward | GGCAATCGATAGGCCACGCTTTCA |
|  | Reverse | AAACTGAAAGCGTGGCCTATCGAT |
| *OsSAP*-GE-3 | Forward | GGCACCACAAGCCAGTTATCCCTG |
|  | Reverse | AAACCAGGGATAACTGGCTTGTGG |
| *OsSAP*-GE-Seq | Forward | CTGCCACAAGCCAGTTATCC |
|  | Reverse | GGTGACAAAGTTAGCCAGATCA |

**
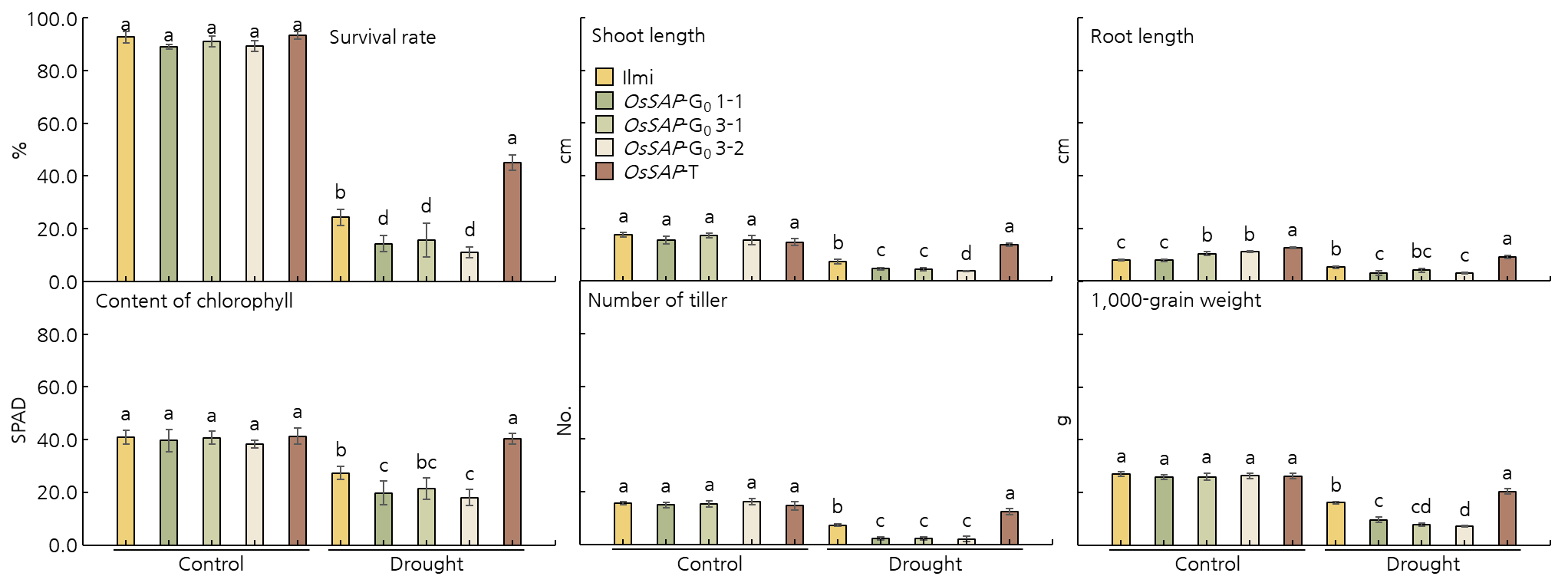
**

**Supplementary Figure S1 ǀ** Investigate of survival rate, shoot length, root length, content of chlorophyll, number of tiller, and 1,000-grain weight after drought stress treatment until the soil moisture content reached 20% at seedling stage. Survival rate, shoot length, root length, content of chlorophyll, number of tiller were investigated when the soil moisture content reached 20%, and 1,000-grain weigh was investigated using seeds harvested after drought stress treatment and re-watering. Evaluated agronomic traits are expressed as means ± SD from five independent biological experiments per line. Bars represent means ± standard error. Means denoted by the same letter are not significantly different (*P* < 0.05) as evaluated by Duncan’s multiple range test.


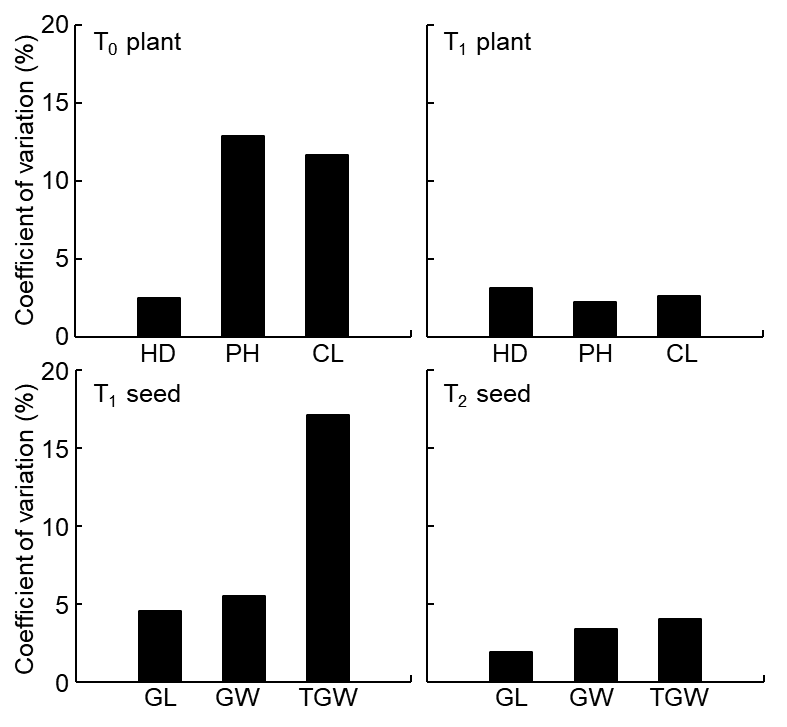


**Supplementary Figure S2 ǀ** Analysis of coefficient of variation (CV) according to plant and seed characteristics in each genome editing generation. In the T_0_ plant, the CV of heading date, plant height, and culm length are 2.5 %, 12.9 %, and 11.7 %, respectively. However, in the T_1_ plant, the CV of heading date, plant height, and culm length are 3.1%, 2.2%, and 2.6%, respectively. In T_1_ plant, since the variation for each trait is very low, the morphological characteristics are fixed and there is a high possibility of homozygous. For T_1_ seed, the CV of grain length, grain width, and 1,000-grain weight were 4.5 %, 5.6 %, and 17.1 %, respectively. However, in T_2_ seed, the CV of grain length, grain width, and 1,000-grain weight are 1.9 %, 3.4 %, and 4.1 %, respectively. Since the variation for each trait in T_2_ seed is very low, it is highly likely to be morphological characteristics are fixed. HD: Heading date, PH: Plant height, CL: Culm length, GL: Grain length, GW: Grain width, TGW: 1,000-grain weight.


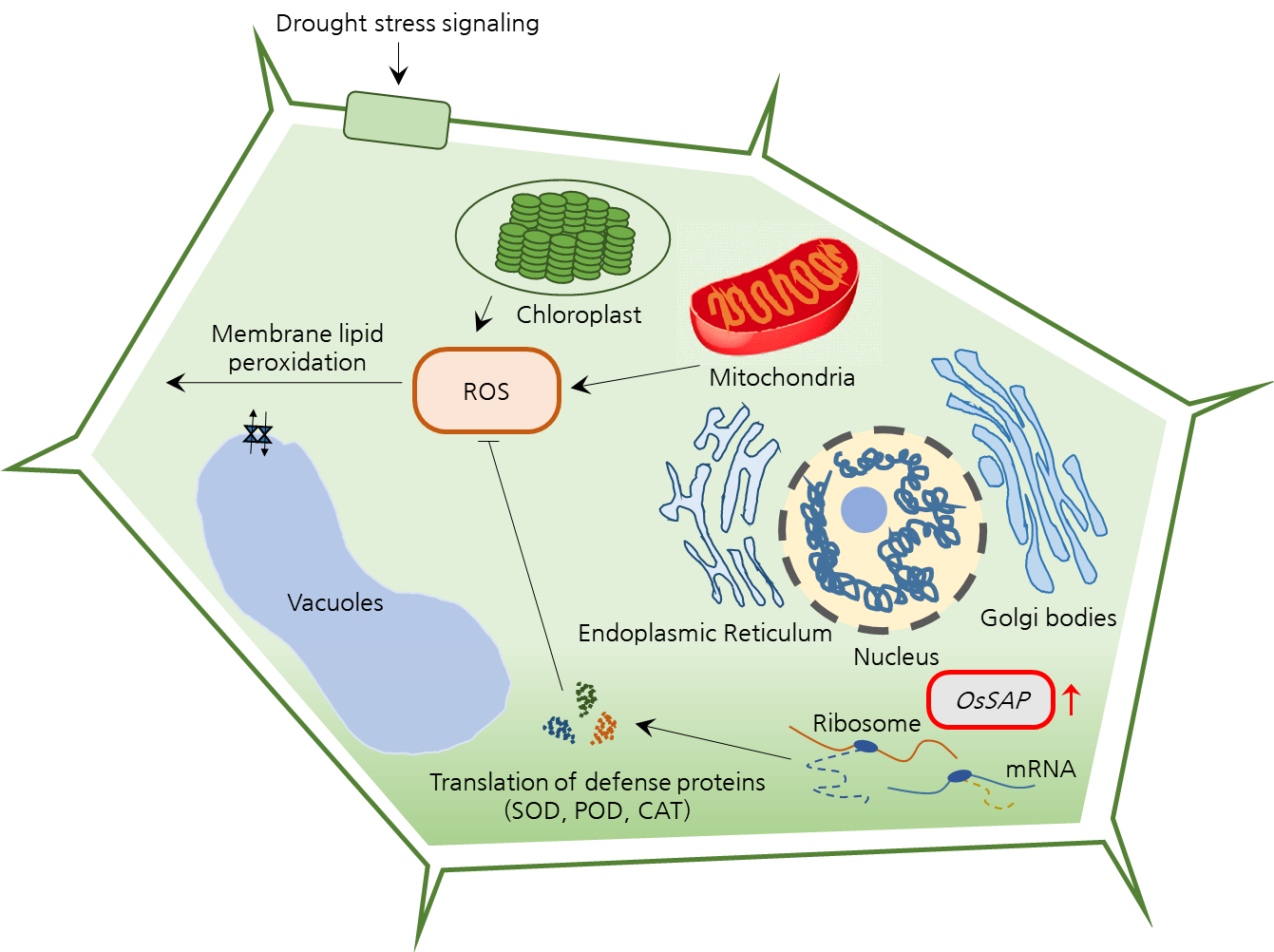


**Supplementary Figure S3 ǀ** Possible model of the control system used by genome editing plant under drought stress. Under drought stress, *OsSAP* is expressed and the activity of the antioxidant enzymes CAT, SOD, and POD increases. These enzymes enhance the scavenging ability of ROS caused by drought stress. Therefore, drought tolerance is improved. In addition, *OsSAP* improves drought tolerance by regulating the expression of stress-related transcription factors under drought stress conditions.
